# The protective role of proton-sensing TDAG8 in the brain injury in a mouse ischemia reperfusion model

**DOI:** 10.1038/s41598-020-74372-7

**Published:** 2020-10-14

**Authors:** Koichi Sato, Ayaka Tobo, Chihiro Mogi, Masayuki Tobo, Nobuhiro Yamane, Masahiko Tosaka, Hideaki Tomura, Dong-Soon Im, Fumikazu Okajima

**Affiliations:** 1 Laboratory of Signal Transduction, Institute for Molecular and Cellular Regulation, Gunma University, Maebashi, 371-8512 Japan; 2 Department of Neurosurgery, Gunma University Graduate School of Medicine, Maebashi, 371-8511 Japan; 3 Laboratory of Cell Signaling Regulation, Department of Life Sciences, School of Agriculture, Meiji University, Kawasaki, 214-8571 Japan; 4 College of Pharmacy, Kyung Hee University, Seoul, 02447 Republic of Korea; 5 Laboratory of Signal Transduction, Faculty of Pharmaceutical Sciences, Aomori University, Aomori, 030-0943 Japan

**Keywords:** Glial biology, Neuroimmunology, Neurophysiology, Neurological disorders

## Abstract

Extracellular acidification in the brain has been observed in ischemia; however, the physiological and pathophysiological implications of the pH reduction remain largely unknown. Here, we analyzed the roles of proton-sensing G protein-coupled receptors, including T-cell death-associated gene 8 (TDAG8), ovarian cancer G protein-coupled receptor 1 (OGR1), and G protein-coupled receptor 4 (GPR4) in a mouse ischemia reperfusion model. Cerebral infarction and dysfunctional behavior with transient middle cerebral artery occlusion (tMCAO) and subsequent reperfusion were exacerbated by the deficiency of TDAG8, whereas no significant effect was observed with the deficiency of OGR1 or GPR4. We confirmed that the pH of the predicted infarction region was 6.5. TDAG8 mRNA was observed in Iba1-positive microglia in the mouse brain. The tMCAO increased the mRNA expression of tumor necrosis factor-α in the ipsilateral cerebral hemisphere and evoked morphological changes in microglia in an evolving cerebral injury. These tMCAO-induced actions were significantly enhanced by the TDAG8 deficiency. Administration of minocycline, which is known to inhibit microglial activation, improved the cerebral infarction and dysfunctional behavior induced by tMCAO in the TDAG8-deficient mouse. Thus, acidic pH/TDAG8 protects against cerebral infarction caused by tMCAO, at least due to the mechanism involving the inhibition of microglial functions.

## Introduction

Brain ischemia or hypoxia depends on several factors that may influence each other to create complex mechanisms for a detrimental outcome. For example, in an ischemic situation, a lack of blood supply causes hypoxia and the inhibition of aerobic respiration and, thereby, increases lactic acid production through glycolysis, causing a decrease in pH from a normal value to 6.1–6.8^1,2^. An acidic pH of around 6.0 is thought to influence mitochondrial function, free-radical formation, synthesis and degradation of cellular components, cell volume control, and endothelial damage; these events lead to irreversible neuronal cell death^3^. A mild acidosis of around pH 6.6, however, does not result in any deterioration of the energy state or morphological evidence of irreversible cell damage^3^. Thus, the pH level may be an important factor in the output of the cellular events during ischemia. However, their mechanisms for signaling an acidic pH in a cerebral injury during ischemia have been poorly elucidated.

Two types of proton-sensing receptors have been reported: ion channels and the OGR1 family of G protein-coupled receptors (GPCRs)^4^. Proton-sensing ion channels include acid-sensing ion channels (ASICs), which sense pH 4–7 depending on the type of the ion channels, and transient receptor potential vanilloid type 1 (TRPV1), which senses pH 4–5^4^. Proton-sensing OGR1-family GPCRs include ovarian cancer G protein-coupled receptor 1 (OGR1), G protein-coupled receptor 4 (GPR4), and T-cell death-associated gene 8 (TDAG8), which sense an acidic pH higher than 6.4^4^. Several studies have shown that proton-sensing GPCRs mediate cellular actions in a variety of cell types^5–8^. OGR1 is expressed in bone cells and smooth muscle cells and has been reported to be involved in a variety of physiologically important responses to extracellular acidification^6,9^. GPR4 has been suggested to induce inflammatory responses in endothelial cells^6,10^. TDAG8 is expressed in lymphoid tissues^11,12^ and has been shown to mediate the inhibition of inflammatory cytokine production in macrophages^7,13,14^ and superoxide anion production in neutrophils^15^.

Proton-sensing GPCRs are also expressed in central nervous system and neural cells. TDAG8 is expressed in cultured microglia from newborn mice and is involved in the acidic pH-induced inhibition of TLR-mediated interleukin-1β (IL-1β) production through cAMP/protein kinase A^16^. Microglia fulfill a central role in the immune system in the brain^17,18^. The activation of microglia and subsequent increase in the synthesis of proinflammatory cytokines, such as IL-1 and tumor necrosis factor-α (TNF-α), have been shown to occur during a stroke^19–23^. IL-1 and TNF-α are known to play a causal role in their neurodegeneration^17,18,24–31^. These results suggest the possibility that microglial TDAG8 in response to ischemic acidification might be involved in brain strokes. On the other hand, GPR4 and OGR1 are expressed in cortical neurons isolated from mouse embryo and N1E115 neuronal cells^6,16,32,33^ and are potential receptors to regulate cellular events in response to brain acidosis.

In the present study, we explored the roles of proton-sensing GPCRs in an ischemic brain model as induced by transient middle cerebral artery occlusion (tMCAO) and reperfusion. We found that a deficiency of TDAG8 exacerbated the dysfunctional behavior and cerebral infarction caused by tMCAO and the following reperfusion. However, the deficiency of OGR1 and GPR4 failed to affect the extent of the infarction under the same tMCAO protocol. Several kinds of experiments, including behavior testing and evaluation of infarction size by TTC stain, Nissl stain, and MRI imaging, suggested that acidic pH/TDAG8 protects against cerebral injury by tMCAO possibly through the mechanism involving inhibitory actions against microglial function.

## Results

### Expression profile of proton-sensing GPCR mRNA in the brain

**Figure 1:**
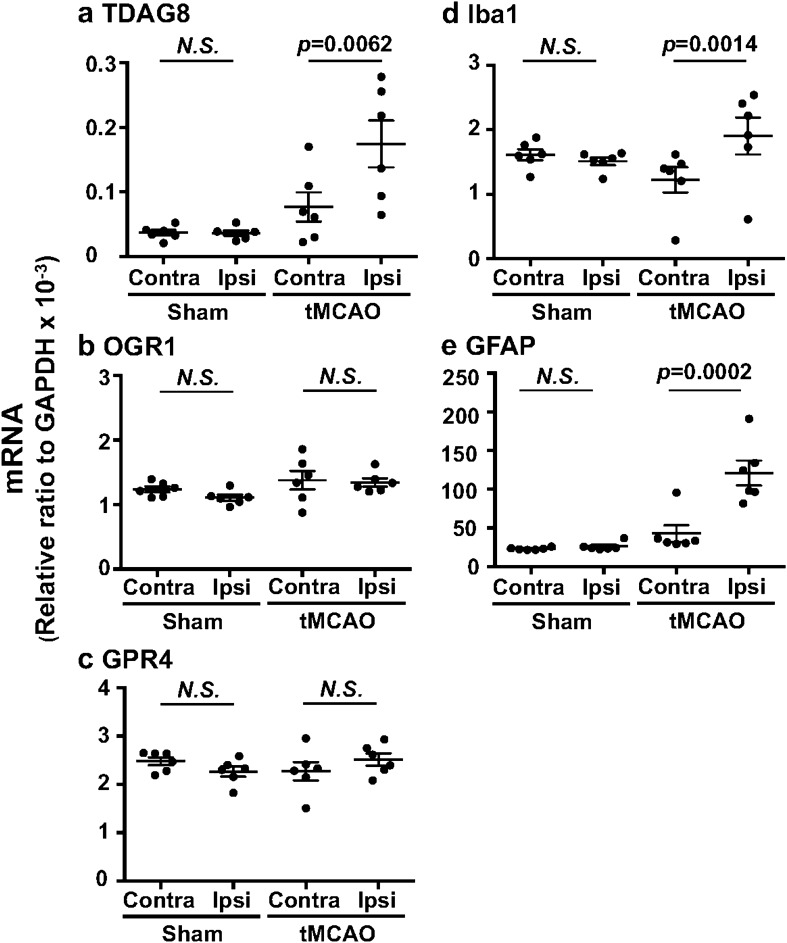
TDAG8 expression was significantly increased in the cerebral hemisphere after the induction of a transient occlusion for the middle cerebral artery (tMCAO). (**a–e**) Quantitative analyses of mRNA expression of TDAG8 (**a**), OGR1 (**b**), GPR4 (**c**), Iba1 (**d**), and GFAP (**e**) in the contralateral (Contra) and ipsilateral (Ipsi) wild-type (WT) hemispheres after the induction of tMCAO for 0.5 h and reperfusion for 24 h. Total RNA was prepared from each cerebral hemisphere of sham (n = 6) and tMCAO mice (n = 6). Results are expressed as the ratio relative to GAPDH. Error bars represent the mean ± SEM. Comparisons between contralateral and ipsilateral hemispheres were assessed using the paired Student’s *t*-test (N.S., not significant). The effect of tMCAO and the following reperfusion is significant (*p* < 0.01).

We first evaluated the mRNA expression of proton-sensing GPCRs under the ischemia. Quantitative mRNA measurement showed that OGR1 and GPR4 are abundantly expressed, as compared with TDAG8, in the mouse brain (Fig. 1). Among proton-sensing GPCRs, however, TDAG8 expression in the ipsilateral hemisphere was significantly higher than in the contralateral hemisphere, which was associated with an increase in the mRNA expression of Iba1 and glial fibrillary acidic protein (GFAP), after the induction of tMCAO for 0.5 h and subsequent reperfusion for 24 h. The expression of OGR1 or GPR4 did not differ between the ipsilateral and contralateral hemispheres. In sham surgery, as expected, no significant change in the mRNA between the ipsilateral and contralateral hemispheres was observed.

### Exacerbation of tMCAO-induced cerebral infarction by TDAG8 deficiency

**Figure 2:**
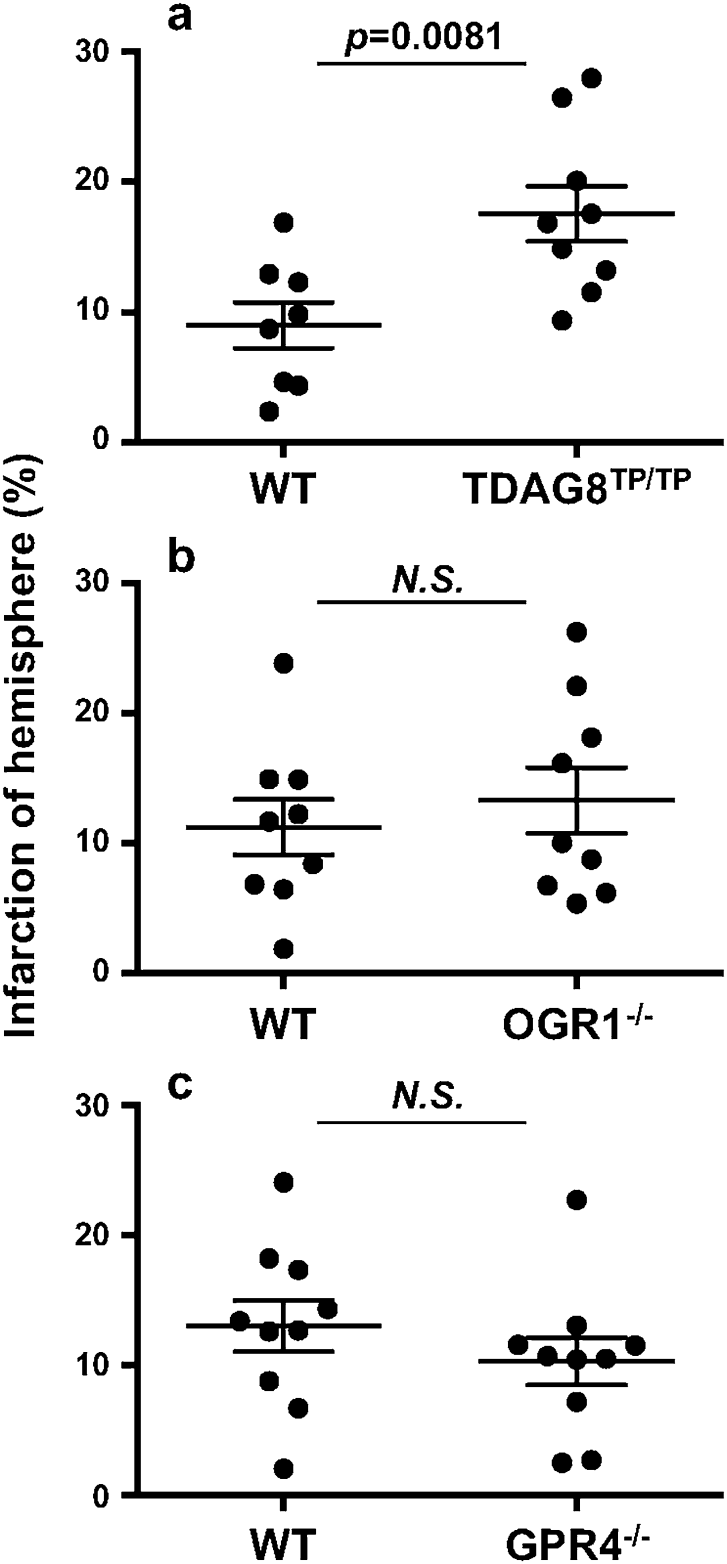
TDAG8 deficiency exacerbated the tMCAO-induced cerebral infarction, whereas no appreciable effect was observed by the deficiency of either OGR1 or GPR4. (**a-c**) Cell damage scores were obtained through analyses of histological Sects. 24 h after the tMCAO/reperfusion. The infarction by the tMCAO in mice deficient in TDAG8 (**a**), OGR1 (**b**), and GPR4 (**c**). Data are shown as the mean ± SEM of each group of WT mice (n = 8 ) and TDAG8-deficient mice (TDAG8^Tp/Tp^, n = 9), WT mice (n = 9) and OGR1-deficient mice (OGR1^−/−^, n = 9), and WT mice (n = 10) and GPR4-deficient mice (GPR4^−/−^, n = 10). Comparisons among groups were assessed using the unpaired Student’s *t*-test. The effect of TDAG8 deficiency was significant (*p* < 0.01). No significant difference was observed between WT and OGR1^−/−^ mice or WT and GPR4^−/−^ mice.

The infarct volume was evaluated for tissue damage using histological staining^21,34^. We first employed cresyl violet staining for Nissl substance in an attempt to see the time-dependent increase in the infarction regions after tMCAO for 0.5 h and reperfusion (Supplementary Information Fig. S2). The infarction area gradually increased depending on the time after reperfusion and expanded from the striatum at 6–24 h to the cortex at 72 h. We performed the tMCAO experiment using a few TDAG8 deficient (TDAG8^Tp/Tp^) mice^7^ and found that the infarction area tended to be increased by the TDAG8 deficiency. To confirm this, we chose the time of 24 h after reperfusion. The infarction area was significantly greater in TDAG8^Tp/Tp^ mice than in WT mice (Fig. 2a). Unless otherwise stated, the tMCAO experiment was performed using this protocol, *i.e.*, 0.5 h tMCAO and subsequent 24 h reperfusion. We next used OGR1-null (OGR1^−/−^) mice^9^ and GPR4-deficient (GPR4^−/−^) mice (Supplementary Information Fig. S1) to examine whether OGR1 and GPR4 are involved in regulation of the injury caused by ischemia. Under the same tMCAO protocol, the deficiency of neither OGR1 nor GPR4 showed any significant effect on the infarction size (Fig. 2b and c). Therefore, TDAG8 seems to play a protective role in the progression of ischemia-induced infarction; however, no evidence of the participation of either OGR1 or GPR4 in the ischemia-induced infarction was detected under our experimental conditions.

**Figure 3:**
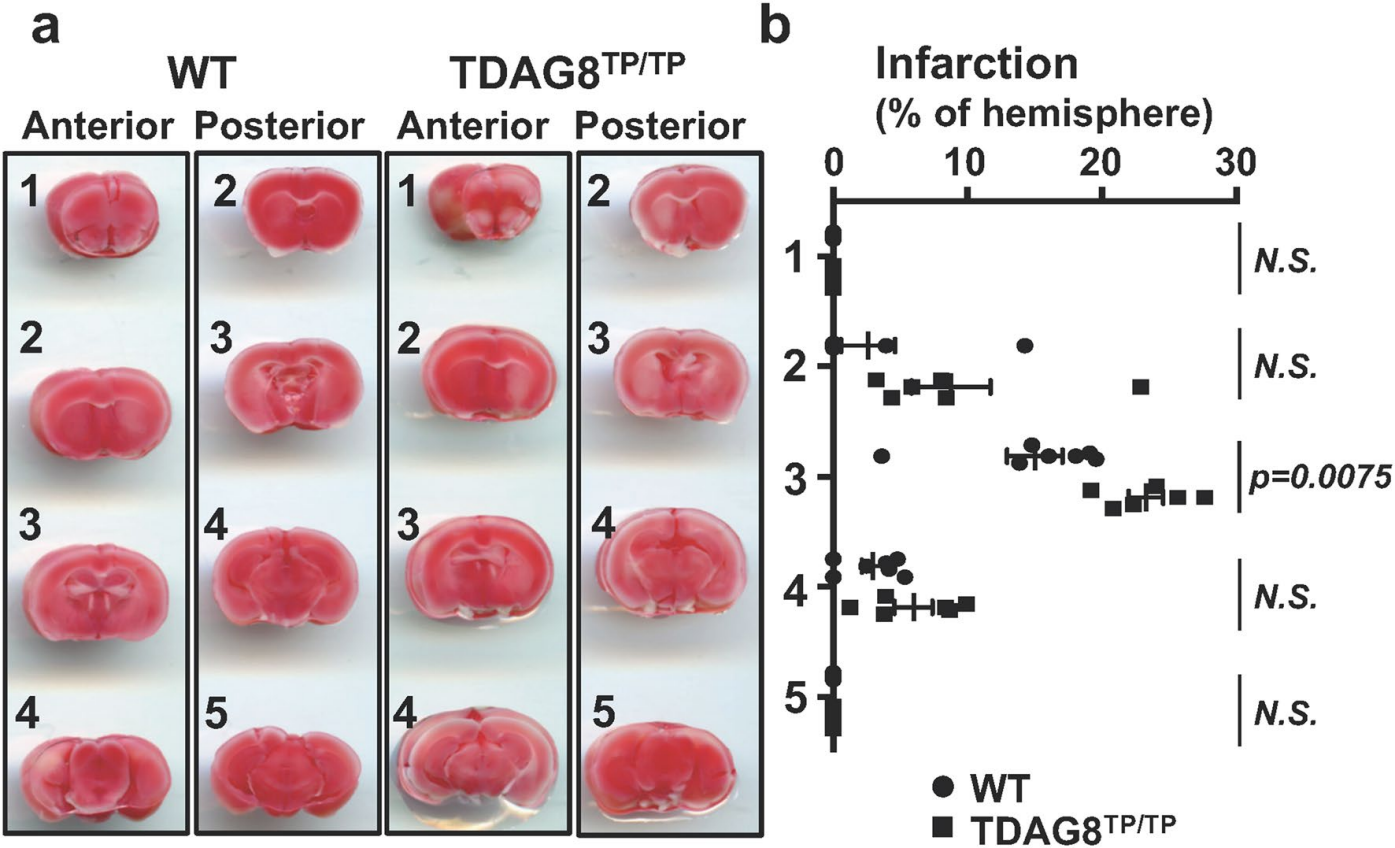
TTC staining showed that TDAG8 deficiency exacerbated the tMCAO-induced cerebral infarction in mouse brains. (**a**) Representative coronal sections of WT and TDAG8^TP/TP^ mice brains stained with TTC 24 h after tMCAO (0.5 h) /reperfusion. The image for both anterior and posterior sides was captured with the scanner. (**b**) The infarct areas were obtained by analyzing coronal sections stained with TTC in WT mice (closed circle, n = 7) and TDAG8^TP/TP^ mice (closed square, n = 6). The infarction (%) was calculated based on the lesion areas of the ipsilateral hemisphere. Error bars represent the mean ± SEM. Comparisons between WT and TDAG8 ^TP/TP^ mice were assessed using the unpaired Student’s *t*-test. (N.S., not significant).

Consistent with these results, severe cerebral damage by tMCAO in TDAG8 mice was also detected by TTC staining (Fig. 3). However, the cerebral damage observed at 24 h after the reperfusion was not sustainable. Thus, the infarcted regions evaluated by TCC staining almost disappeared in TDAG8-deficient mice as well as in WT mice one month after the reperfusion. These results suggest that the neural ability to regenerate overcomes the damage due to the TDAG8 deficiency, at least in our experimental protocol of 0.5 h tMCAO (Supplementary Information Fig. S3). To further confirm the protective role of TDAG8 in the ischemia-induced neurological deficits, we performed experiments to measure behavioral function as previously described^35^. To show the deficit level, pictures of level 1 and level 3 are included as examples (Supplementary Information Fig. S4b and c). Consistent with the results of the infarction (Fig. 2a), the damage was unchanged in the heterozygous TDAG8 (TDAG8^WT/Tp^) mice but was significantly exacerbated in the homozygous TDAG8^TP/TP^ mice (Supplementary Information Fig. S4a).

### Lack of influence by the TDAG8 deficiency on regional cerebral blood flow and pH

The regional cerebral blood flow during surgery for MCAO was monitored via laser Doppler flowmetry, which showed no obvious difference between WT and TDAG8^Tp/Tp^ mice (Supplementary Information Fig. S5). Thus, TDAG8 does not seem to affect the vascular functions to regulate blood flow before and after the occlusion of the artery.

**Figure 4:**
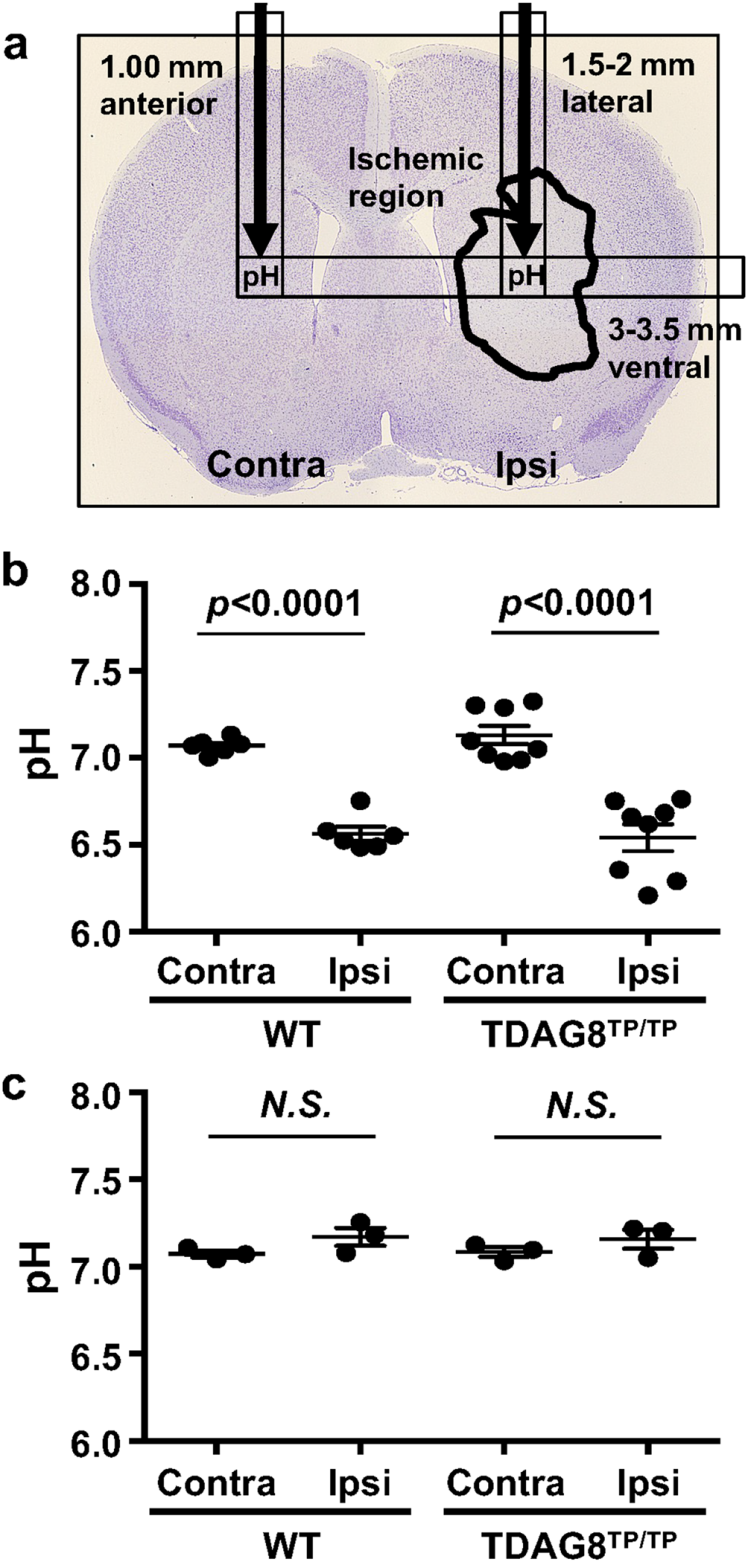
The pH declines in the ischemic region as a result of the tMCAO independently of TDAG8 deficiency. (**a**) Localized pH changes were measured using a pH microsensor in the ipsilateral and contralateral regions of interest (1.5–2 mm lateral, 3–3.5 mm ventral, and 1 mm anterior to the bregma). The predicted position of the pH sensor is indicated by a black arrow on the histogram image of Nissl staining for cell damage 24 h after tMCAO for 0.5 h and reperfusion. (**b**) Acidification of the ischemic region after MCAO for 0.5 h in WT mice (n = 6) and TDAG8^Tp/Tp^ mice (n = 8). Error bars represent the mean ± SEM. Comparisons between contralateral and ipsilateral hemispheres were assessed using the paired Student’s *t*-test. The effect of MCAO was significant (*p* < 0.01, Contra vs. Ipsi). (**c**) Restoration of acidic pH in the ischemic region 24 h after the tMCAO/reperfusion in WT mice (n = 3) and TDAG8^Tp/Tp^ mice (n = 3). Error bars represent mean ± SEM. Comparisons between contralateral and ipsilateral hemispheres were assessed using the paired Student’s *t*-test (N.S., not significant).

To measure the pH, we inserted a pH sensor at a position in the predictable infarction region 0.5 h after tMCAO (Fig. 4a) and confirmed the previous results^1,2^, which were obtained using a method different from ours, that the extracellular pH in the ipsilateral and contralateral regions of interest were around 6.5 and 7.1, respectively (Fig. 4b). The extracellular pH change was also observed in prolonged (permanent) ischemia of at least 5 h (data not shown). The acidic pH in the ipsilateral region was restored to the level of the contralateral region at 1 h (data not shown) and 24 h (Fig. 4c) after the reperfusion. Importantly, the extracellular pH changes during occlusion and after reperfusion were not appreciably affected by the TDAG8 deficiency. This suggests that changes in the infarct volume in the TDAG8-deficient mice reflect downstream events of the pH change and not simply changes in the magnitude of the original injury.

### TDAG8 mRNA expressed in the Iba1-labeled microglia

**Figure 5:**
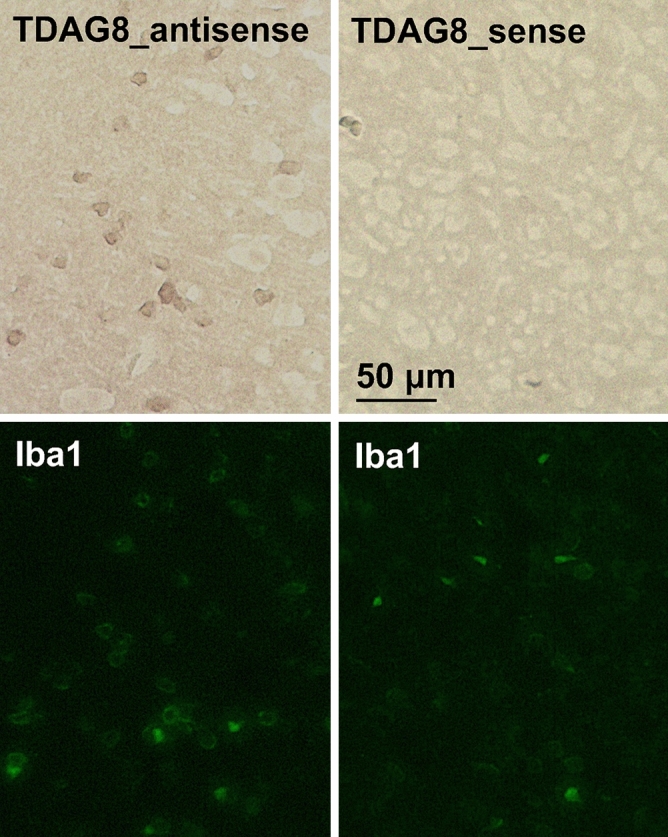
TDAG8 mRNA is expressed in Iba1-labeled cells. A digoxygenin-labeled antisense RNA probe specific to TDAG8 mRNA was visualized by in situ hybridization in a mouse brain (brown). The slices were subsequently stained with an anti-Iba1 antibody (Alexa Fluor 488, green). There was no apparent signal in the control sections according to the sense probes. Scale bar is 50 μm. Results shown are the representatives of two separate experiments.

As shown in Fig. 1, the TDAG8 mRNA expression is higher in the ipsilateral region than in the contralateral region, which is associated with enhancement of the Iba1 mRNA expression. To examine the expression profile of TDAG8 at the cellular level in a normal mouse brain, we performed in situ hybridization and observed strong TDAG8 mRNA signals throughout in the cortical and striatum areas. The result showed that almost all of the TDAG8 mRNA-expressing cells were labeled with Iba1 (Fig. 5).

### Involvement of TDAG8 in the regulation of cytokine expression in cerebral injury

We previously reported that extracellular acidification inhibited LPS-induced IL-1β production in isolated microglia^16^. Similarly, not only the IL-1β production but also the TNF-α production induced by LPS was downregulated by acidic pH in a manner dependent on TDAG8 in isolated microglia (Supplementary Information Fig. S6). There was no significant influence on the production of cytokines at neutral pH of around 7.4, regardless of TDAG8 deficiency. An extracellular acidic pH from 7.2 to 6.4 clearly reduced the LPS-induced production of either IL-1β or TNF-α, and their inhibition was dependent on TDAG8. Thus, the TDAG8 deficiency significantly reversed the inhibitory activities induced by a mild-acidic pH of 7.2 to 7.0 for the IL-1β production and a pH of 7.2 to 6.4 for the TNF-α production in isolated microglia (Supplementary Information Fig. S6). It should be noted, however, that the inhibition of cytokine production at pH 6.4, when the pH level was attained by tMCAO in the ipsilateral region of the living mouse (Fig. 4b), was still sensitive to TDAG8 for TNF-α but not for IL-1β. The mechanism of TDAG8-independent inhibition of cytokine production by acidic pH remains to be established.

**Figure 6:**
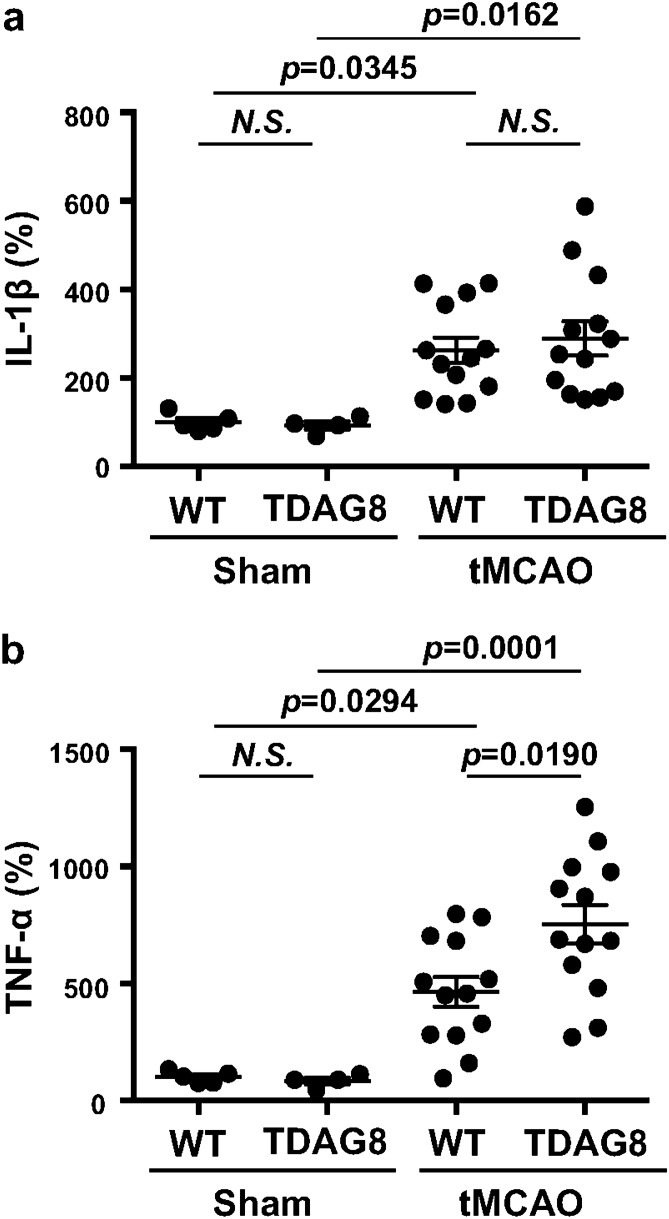
TDAG8 deficiency enhanced the mRNA expression for TNF-α in the infarction regions induced by tMCAO. (**a, b**) RNA was prepared from the cerebral hemisphere 24 h after tMCAO/reperfusion or sham operation. The mRNA expressions of IL-1β (**a**) and TNF-α (**b**). Results are expressed as mRNA expression of the ipsilateral vs. contralateral hemisphere. Data are shown as the mean ± SEM of sham mice (WT n = 5, TDAG8TP/TP n = 4) and tMCAO mice (WT n = 13, TDAG8TP/TP n = 13). Comparisons among groups were assessed using a two-way ANOVA followed by the Tukey test for multiple-group comparisons (N.S., not significant). The tMCAO appreciably induced mRNA expressions of cytokines compared to the sham surgery (*p* < 0.01). The effect of TDAG8 deficiency for on TNF-α expression is significant (*p* < 0 .05).

The results described above imply that TDAG8 might be involved in the cytokine response to the tMCAO/reperfusion. Therefore, we measured the level of cytokine mRNA in total RNA extracts from the ipsilateral and contralateral cerebral hemispheres 24 h after the tMCAO/reperfusion (Fig. 6). In sham surgery, the expression of cytokine mRNA did not differ between the ipsilateral and contralateral hemispheres. The tMCAO appreciably increased the expression of either IL-1β or TNF-α mRNA in the ipsilateral hemisphere as compared with the contralateral hemisphere. As expected, the expression of TNF-α mRNA was significantly enhanced by the TDAG8 deficiency (Fig. 6b). The expression of IL-1β mRNA, however, tends to increase, the effect of the TDAG8 deficiency was not significant (Fig. 6a). The lack of a significant effect on IL-1β might be related to the pH level in the ipsilateral region, which was lower than the TDAG8-dependent range (Fig. 4b and Supplementary Information Fig. S6). Thus, TDAG8 can sense an acid environment from a neutral to mildly acidic pH of around 6.4 as induced by MCAO and downregulate the production of cytokine, at least TNF-α, in the injury.

### Potential suppressive role of TDAG8 in microglial activation in an evolving cerebral injury

**Figure 7:**
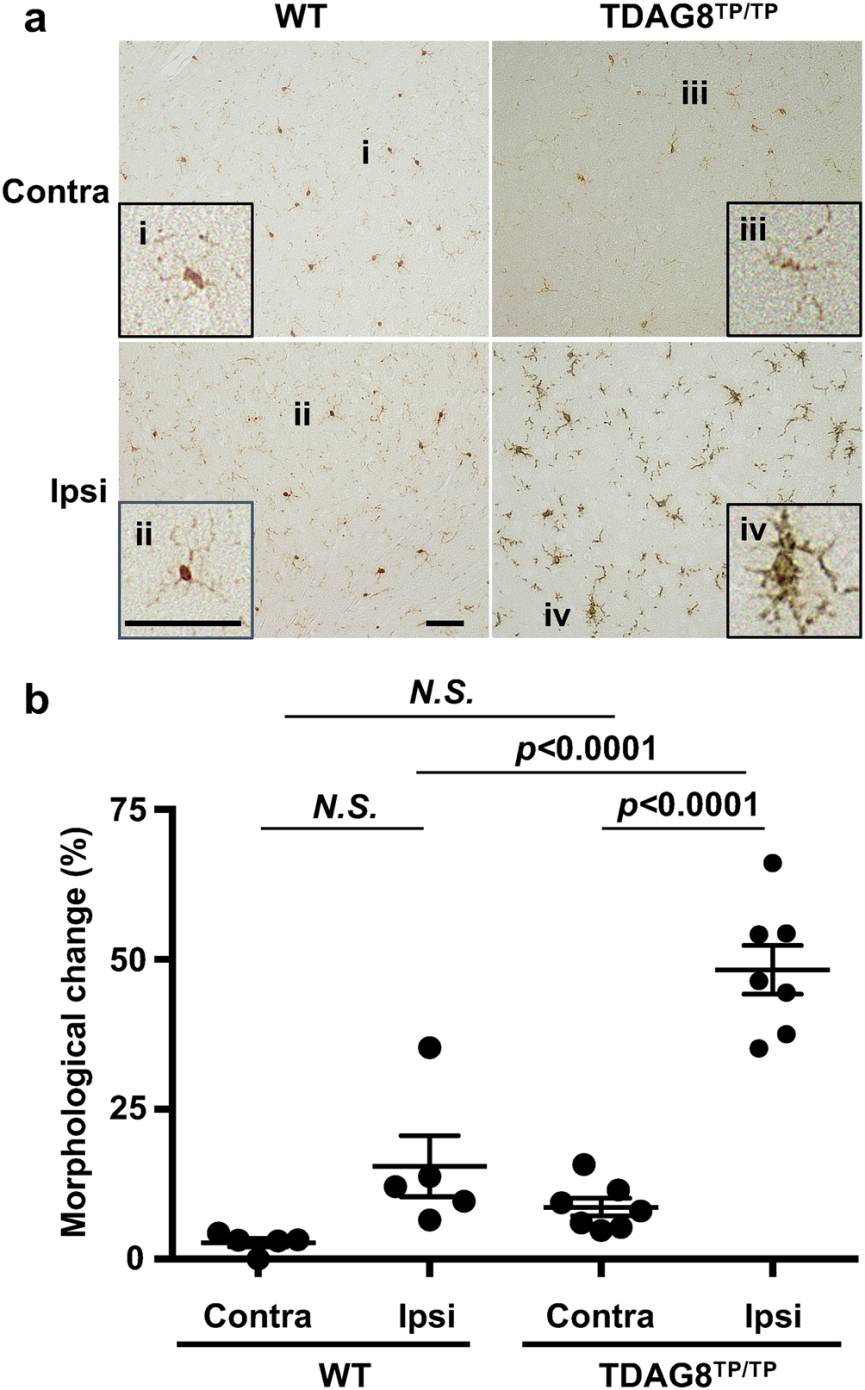
TDAG8 regulates the morphological change in microglia in the somatosensory area near the infarcted regions by tMCAO. The morphological change in microglia in the somatosensory area of the coronal section at 1 mm anterior to the bregma was measured in mice after tMCAO for 0.5 h and reperfusion for 24 h. (**a**) Typical images of Iba1-labeled cells (brown, DAB) from the ipsilateral and contralateral regions in WT and TDAG8^Tp/Tp^ mice are shown. Inserts show higher magnification of the representative cells. (**b**) The cells with ramified or amoeboid-like morphology were visually counted (20–50 cells), and the morphological change was expressed as a percentage of amoeboid-like cells among total cells. Data are shown as the mean ± SEM of WT mice (n = 5) and TDAG8^TP/TP^ mice (n = 7). Comparisons among groups were assessed using a two-way ANOVA followed by the Tukey test for multiple-group comparisons (N.S., not significant). The Iba1-labeled cells with retracted and stout processes were apparently induced by the tMCAO in TDAG8^Tp/Tp^ mice (*p* < 0.01), and the effect was not significant in WT mice.

Microglial activation in the brain has also been observed as morphological changes in relation to an evolving cerebral injury in ischemic stroke^24,36,37^. The ipsilateral hemisphere contains three regions, the core, the peri-infarct, and the healthy region, distal to the developing brain lesion. In the healthy region, resting microglia exhibit ramified morphology with a small soma and long, thin, primary processes. Injury induces soma enlargement and process retraction of the microglia, which then become amoeboid, rod-like, and giant cells in the peri-infarct and core regions. As shown in Supplementary Information Fig. S2b, the infarction had expanded from the striatum to the cortex depending on the reperfusion time after tMCAO. Therefore, we analyzed whether microglial activation with morphological change occurs in the somatosensory area of the ipsilateral hemisphere as a result of tMCAO/reperfusion (Fig. 7). Microglia with ramified shapes were dominantly observed in the contralateral cortex regardless of the presence or absence of TDAG8 (i and iii). In the ipsilateral cortex, a cell with an enlarged soma and short processes was occasionally observed, although the major cell types showed ramified morphology in WT mice (ii). The tMCAO-induced morphological change in the ipsilateral region was clearly observed with the TDAG8 deficiency. Thus, the microglia with an enlarged soma and stout processes are significantly increased in TDAG8^Tp/Tp^ mice (iv). These results suggest that TDAG8 is a suppressive regulator of microglial activation in the injury.

### Transient MCAO pathogenesis attenuated by minocycline in a mouse model

**Figure 8:**
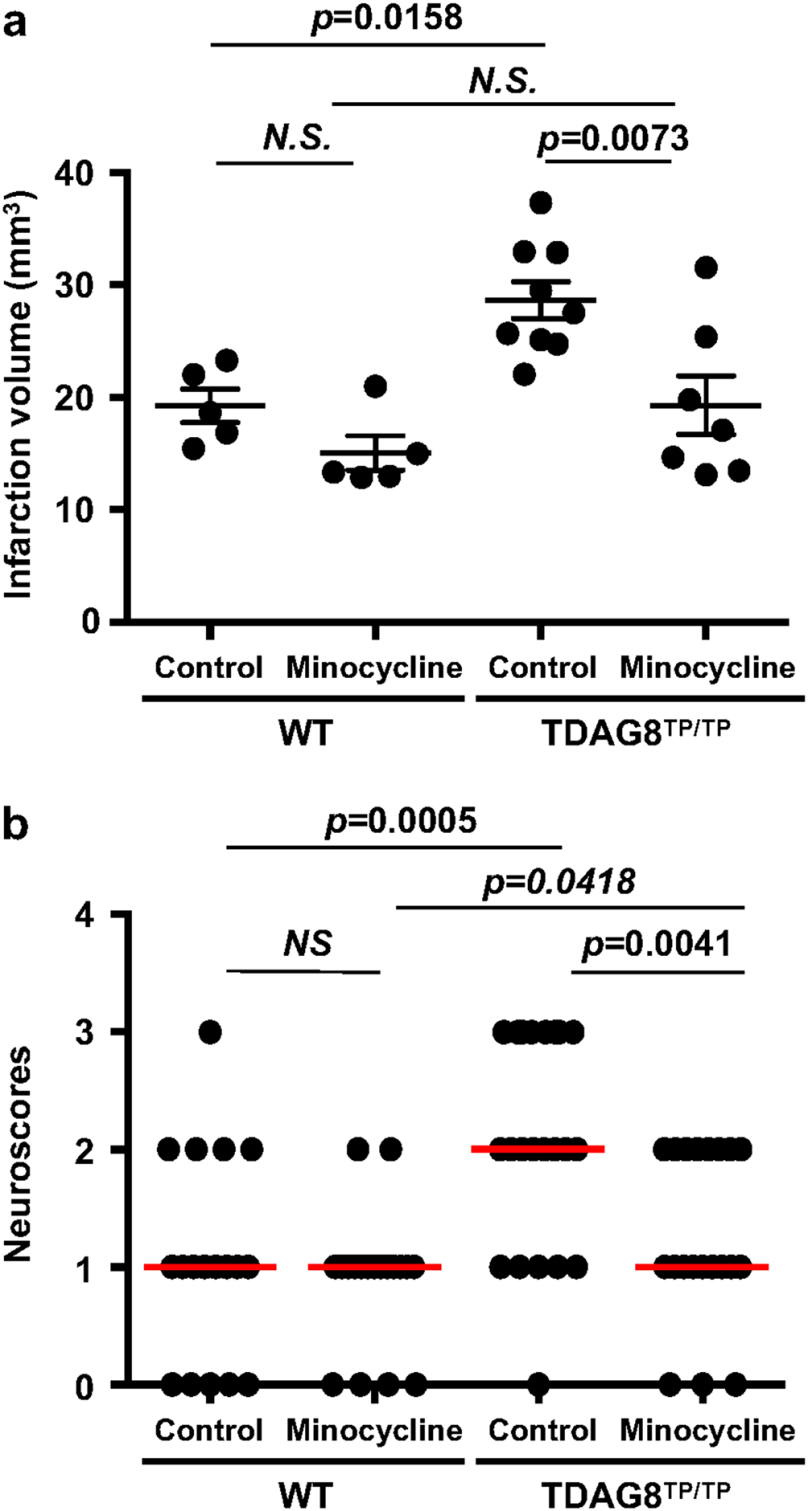
Effects of minocycline on the cerebral infarction and neurological deficit. Mice (WT and TDAG8^TPTP^) were treated with minocycline or vehicle. After the mice were subjected to tMCAO/reperfusion, the head MRI_T2 enhanced image was evaluated. (**a**) Effect of minocycline on infarct size in tMCAO of WT mice (Control n = 5, Minocycline n = 5) and TDAG8^Tp/Tp^ mice (Control n = 9, Minocycline n = 7 ). Error bars represent the mean ± SEM. Comparisons among groups were assessed using a two-way ANOVA followed by the Tukey test for multiple-group comparisons (N.S., not significant). The lesion is more severe in TDAG8^Tp/Tp^ mice than in WT mice (*p* < 0.01). The effect of minocycline on infarction size is significant in TDAG8^Tp/Tp^ mice (*p* < 0.01) but not in WT mice. (**b**) Effect of minocycline on neurological scores in tMCAO of WT mice (Control n = 19, Minocycline n = 19) and TDAG8^Tp/Tp^ mice (Control n = 24, Minocycline n = 24). Data are shown as neurological scores (closed circle) and median (red line). Comparisons among groups were assessed using the unpaired Mann–Whitney test (N.S., not significant). Minocycline significantly reduced neurological impairment induced by tMCAO in TDAG8^Tp/Tp^ mice (*p* < 0.01) but not in WT mice.

An antibiotic minocycline has been reported to delay disease onset and progression in mouse ischemia models in association with an inhibition of glial cell activation^38–41^. As shown in Supplementary Information Fig. S7, minocycline suppresses the LPS-activated production of inflammatory cytokines, including TNF-α, IL-1β, and proIL-1β, in isolated microglia. On the other hand, the LPS-induced cytokine and VCAM-1 expression were not affected by the minocycline treatment in astrocytes. Thus, the drug showed an anti-inflammatory property for microglia but not for astrocytes at least in vitro*.* To assess the effects of minocycline in vivo, minocycline was intraperitoneally administered four times: once daily beginning 3 days before and immediately after the tMCAO/reperfusion. As shown in Supplementary Information Fig. S8, the infarction in living mice can be assessed using an MRI device 24 h after the surgery. The MRI data showed that the infarction volume was greater in TDAG8^Tp/Tp^ mice than in WT mice (Fig. 8a), which is in good agreement with the results of staining with TTC and cresyl violet. The severe infarction in the TDAG8^Tp/Tp^ mice was significantly reduced by the minocycline treatment. In WT mice, it tends to decrease, but the effect of the agent was not significant. Consistent with the result for infarction size, the neurological damage was more severe in TDAG8^Tp/Tp^ mice than in WT mice (Fig. 8b). Minocycline did not appreciably affect the neurological scores in WT mice but significantly decreased the exacerbated damage in TDAG8^Tp/Tp^ mice to the level of that in WT mice. Thus, minocycline treatment attenuated the infarction size and neurological impairment in TDAG8^Tp/Tp^ mice. These results suggest that TDAG8 may have a protective and inhibitory function against cerebral infarction caused by tMCAO, possibly through the mechanism involving inhibitory actions against some microglial functions.

## Discussion

Brain acidosis, along with hypoxia, is a hallmark of acute brain damage, such as ischemia and traumatic injury. Proton-sensing ion channels, such as ASICs and TRPV1, have been suggested to be involved in acidosis-induced neuron cell death^42^. In addition to ion channels, the brain expresses an abundant level of proton-sensing GPCRs, including TDAG8, OGR1, and GPR4, which sense an acidic pH of higher than 6^5,6^. However, the cellular events and their mechanisms in the central nervous system in response to the mildly acidic condition are largely unknown. In the current study, we focused on the role of proton-sensing GPCRs in brain injuries after the ischemia and found that TDAG8 plays a protective role in the progression of ischemia-induced infarction possibly through the mechanism involving changes in microglial functions.

First, the infarction size in the present tMCAO experiment was significantly greater in the TDAG8^Tp/Tp^ mice than in the WT mice (Fig. 2a and 8a). Moreover, the ischemia-induced neurological deficit as evaluated by scoring the behavioral function was much greater in the TDAG8^Tp/Tp^ mice than in the WT mice (Fig. 8b). Thus, ischemia-induced cerebral infarction and dysfunctional behavior were exacerbated by TDAG8 deficiency. In the present occlusion model, we found that a tissue acidification of pH 6.5 was observed in the predicted ipsilateral core during ischemia for 0.5 h, then was restored to a normal pH of 7.1 at 24 h after reperfusion (Fig. 4). The pH change was not influenced by TDAG8 deficiency. Needless to say, the infarction size is affected by the cerebral blood flow. However, the cerebral blood flow before, during, and after occlusion of the artery was not affected by the gene deficiency, suggesting that vascular functions to regulate the blood flow do not seem to be appreciably affected by the lack of a proton-sensing receptor. Thus, tMCAO causes the tissue acidification, which is sufficient to stimulate TDAG8, in living tissue. These results suggest that acidic pH-stimulated TDAG8 works as a protective receptor for the ischemia-induced brain injury in vivo, possibly through a different mechanism from the change in the vascular system.

Second, the implication of microglial involvement in the regulation of ischemia-induced infarction was supported by several observations. TDAG8 mRNA is abundantly expressed in isolated microglia, whereas the mRNA of either GPR4 or OGR1 is very low or undetectable^16^. In situ hybridization experiments showed that the major cell type expressing TDAG8 in the brain is the microglia (Fig. 5). A mildly acidic pH up to around 6.4 was able to inhibit the LPS/TLR-mediated TNF-α production in a manner dependent on TDAG8 in isolated microglia (Supplementary Information Fig. S6) and the TDAG8 deficiency enhanced the expression of TNF-α production in the evolving cerebral injury in vivo (Fig. 6). Moreover, the morphological change from resting ramified microglia to activated microglia was significantly increased in the somatosensory area of the ipsilateral hemisphere obtained from TDAG8^Tp/Tp^ mice as compared with that obtained from WT mice (Fig. 7). Thus, acidosis or an extracellular acidic pH, through TDAG8, seems to inhibit microglial activation in terms of inflammatory cytokine production and morphological change. Supporting this, extracellular acidification inhibited the migration of microglia^43^ and store-operated Ca^2+^ influx^44^. On the other hand, hypoxia, another aspect of brain ischemia, has been shown to potentiate microglial activation and have detrimental effects on the nervous system^45^. Thus, hypoxia and acidosis, hallmarks of acute brain damage, seem to act on microglia in opposite ways; hypoxia, possibly through HIF-1, activates the microglia and induces inflammatory cytokine production, whereas mild acidosis inhibits the activation of the cells. TDAG8 may mediate the acidosis-induced inhibitory effect on hypoxia-induced microglial activation.

Finally, the anti-inflammatory effect of minocycline was confirmed in the isolated microglia; thus, the antibiotic suppressed the LPS-activated production of inflammatory cytokines in the cells, but not in isolated astrocytes (Supplementary Information Fig. S7). Although the administration of minocycline did not significantly improve the cerebral infarction or dysfunctional behavior in WT mice, the antibiotic significantly improved these ischemia-induced neurological damages in TDAG8^Tp/Tp^ mice (Fig. 8).

While our results support the idea that the TDAG8 expressed in the microglia senses the acidic state of the ischemic region and plays a critical role in the recovery after ischemia-induced brain damage, the involvement of other resident cells, such as astrocytes, and infiltrated cells, such as neutrophils and macrophages, in the brain during ischemia cannot be ruled out^19,22,23^. An increased expression of GFAP is known to be due to the process of astrogliosis with astrocyte activation in the infarction core^22^. In our ischemia reperfusion model, the expression of Iba1 mRNA nearly doubled in the ipsilateral hemisphere together with GFAP (Fig. 1e). However, the isolated astrocyte fraction did not show any detectable response to the acidic pH^16^. We tentatively speculate that microglia accumulate and/or proliferate at the lesion site and become activated, which induces astrogliosis. TDAG8 is also profoundly expressed in mouse peritoneal macrophages and human neutrophils and regulates, in a suppressive manner, the acidic pH-induced inhibition of cellular responses, including inflammatory cytokine production^7^ and superoxide anion production^15^. Microglia are considered to be the main source of inflammatory cytokines at acute time points after injuries such as tMCAO^18^. However, infiltrated-peripheral inflammatory cells containing macrophages have been reported in the ischemic brain^21^. Thus, infiltrated peripheral blood leukocytes may exert some influence on the exacerbation of brain injury in TDAG8^Tp/Tp^ mice.

Proton-sensing GPCRs other than TDAG8 are also expressed rather abundantly in the brain; GPR4 and OGR1 are expressed in cortical neurons, and GPR4 is expressed in endothelial cells^6,16,32^. In N1E-115 neuronal cells, extracellular acidification induced Ca^2+^ mobilization in association with cGMP regulation through OGR1 and activated the PI3K/Akt pathway via uncharacterized mechanisms^32^. GPR4 is usually coupled to the G_s_/cAMP signaling pathway^5^. Akt, Ca^2+^ and cAMP signaling pathways have been known to be critical for neuronal cell activities including neuronal cell survival, neurite extension, and neuronal glucose homeostasis. Thus, OGR1 and GPR4 seem to be involved in beneficial neuronal cell activities. On the other hand, acidosis has been reported, through GPR4, to increase the expression of inflammatory genes such as chemokines, cytokines, adhesion molecules, NF-κB pathway genes, COX-2, and stress-response genes in vascular endothelial cells^6,10^ and the brain endothelial cell line, bEND.3 cells (preliminary results), suggesting that GPR4 is involved in the penetration of inflammatory cells into brain lesion sites. Thus, OGR1 and GPR4 may sense the acidic environment induced by MCAO and participate in brain pathophysiology. However, we could not detect any evidence of the participation of either OGR1 or GPR4 using our tMCAO/reperfusion protocol (Fig. 2b and c). In conclusion, we demonstrated that TDAG8, under an acidic environment, has possibly neuroprotective effects on cerebral ischemia through the mechanisms involving change in the functions of resident microglia and partly invaded macrophages. Although further studies are necessary to clarify the roles of proton-sensing GPCRs in brain functions after ischemic injury, they may help to identify the therapeutic targets for a brain injury accompanied by acidosis.

## Methods

### Materials

Anti-actin antibody (A5060), cresyl violet acetate, lipopolysaccharide (LPS, L3012), minocycline hydrochloride (M9511), and 2,3,5-triphenyltetrazolium chloride (TTC) were purchased from Sigma-Aldrich (St. Louis, MO, USA); Mouse TNF-α ELISA kit (DuoSet), Mouse IL-1β ELISA kit (DuoSet), anti-mouse TNF-α antibody (AB-410-NA), anti-mouse IL-1β (AB-401-NA) antibody, and anti-mouse vascular cell adhesion molecule-1 (VCAM-1, AF643) antibody were from R&D Systems (Minneapolis, MN, USA); BCA Protein Assay was from Thermo (Rockford, IL); anti-CD11b antibody (M170.15) was from Acris Antibodies (Herford, Germany); anti-F4/80 antibody (BM8) was from Sanbio BV (Uden, The Netherlands); anti-glial fibrillary acidic protein (GFAP) antibody was from PROGEN (Heidelberg, Germany), and fatty acid-free bovine serum albumin (BSA, Fraction V) was from Calbiochem-Novabiochem Co. (San Diego, CA, USA); anti-Iba1 antibody (019–19741) was from Wako Chemicals (Osaka, Japan); anti-rabbit Alexa Fluor 488 conjugated secondary antibody from Life Technologies (Carlsbad, CA); and quantitative real-time PCR (RT-qPCR) probes specific for TNF-α (Mm00443258), IL-1β (Mm01336189), TDAG8 (Mm00433695), GPR4 (Mm00558777), OGR1 (Mm01335272), Iba1 (Mm00479862), GFAP (Mm01253033), and glyceraldehyde 3-phosphate dehydrogenase (GAPDH, 4352932E) were from Applied Biosystems (Foster City, CA, USA). TDAG8-deficient mice (TDAG8^Tp/Tp^) was kindly provided by Drs K. Horie and J. Takeda of Osaka University (Osaka, Japan), Dr. Takao Shimizu of Tokyo University (Tokyo, Japan), and Dr. Satoshi Ishii of Akita University (Akita, Japan). The sources of all other reagents were the same as described previously^7,9,13,16^.

### Mice

All animal experiments were conducted according to the animal committee’s guidelines for animal care and use, and the study was approved by the animal committee of Gunma University (Permit Numbers 14–29 and 18–13). The mice were maintained in sterile cages on sterile bedding and housed in rooms at a constant temperature and humidity. Sterile food and water were fed to the mice ad libitum. TDAG8^Tp/Tp^ mice were obtained by backcrossing to C57BL/6 mice more than eight generations from TM88ICR mice, which contain a transposon insertion in the *tdag8*^46^. Offspring with a single transposon inserted into the *tdag8* were identified by PCR-genotyping^7^. OGR1-null (OGR1^−/−^) mice were generated as described previously^9^. GPR4-deficient (GPR4^−/−^) mice were generated as shown in Supplementary Information Fig. S1a, and a large fragment containing exon 2 of the *gpr4* gene and its downstream 7.0 kb fragment was obtained by PCR from the 129/Sv mouse BAC genomic library and subcloned into a pBlueScript II vector. The cassette containing the β-galactosidase/neomycin phosphotransferase fusion gene was inserted in the targeting vector replacing the coding sequence in *gpr4* exon 2 (ATG to Bcl I site). The targeting vector was linearized by Not I and electroporated into 129/Sv embryonic stem cells. Neomycin-resistant ES clones were screened for homologous recombination by Southern blot (Supplementary Information Fig. S1b). PCR genotyping for GPR4 deficiency was performed with genomic DNA from tail tips using the primers (Supplementary Information Fig. S1c). Positive ES clone (J8) cells were injected into C57BL/6 blastocysts to generate chimeric mice. The mouse was outcrossed with C57BL/6 mice more than eight times. Wild-type (WT) and gene-deficient (TDAG8^Tp/Tp^, OGR1^−/−^, GPR4^−/−^) mice were maintained by heterozygous brother-sister mating. Neither TDAG8 nor OGR1-deficient mice showed any appreciable phenotype change as compared with their littermate WT mice^7,9^. For example, age-dependent change in body weight and offspring number were hardly affected by their gene deficiency. As for GPR4-deficient mice, the earlier study reported that GPR4-null adult mice appeared phenotypically normal; however, a fraction of the knockout embryos and neonates had spontaneous hemorrhages and defective vascular muscle cell coverage^47^. On the other hand, GPR4-deficient mice used in the present study did not show any abnormal change in offspring number or body weight. Thus, none of the TDAG8, OGR1, or GPR4-deficient mice used in the present study seem to have an obviously abnormal vascular system, at least before occlusion of the vessels. In fact, as shown later, there was no detectable change in cerebral blood flow between the WT and the TDAG8-deficient mice. Male C57BL/6 10 weeks of age were used for surgery to occlude the origin of the middle cerebral artery (MCAO). C57BL/6 pups 1 to 2 days old were also generated to prepare glial cells.

### Surgery for ischemia, measurement of pH, and monitoring of cerebral blood flow

The MCAO was essentially performed essentially as described previously^34,48^ using a 6–0 silicon-coated monofilament suture (Doccol Corporation #6021910). Briefly, anesthesia was induced by inhalation of 2% isoflurane and maintained via inhalation of 1.5% isoflurane. The body temperature of the mice during surgery was maintained with a heating plate. Under a stereomicroscope, the left common carotid artery (CCA), the bifurcation of the internal common carotid artery (ICA) and external common carotid artery (ECA) were carefully dissected from surrounding tissue via a midline pretracheal incision. A small hole was made in the ECA between the permanent and temporary sutures. The 6–0 silicon-coated monofilament suture was inserted into the ECA. The monofilament suture was gently advanced from the lumen of the ECA into the ICA for a distance of 9–10 mm beyond the bifurcation of the CCA to occlude the origin of the MCA. The 7–0 silk suture on the ECA was tightly tied to fix the monofilament suture in position. The mice in the cage were placed under a heating lamp on the heating plate during the post-surgery period (0.5 h). For transient MCAO (tMCAO), the mouse was anesthetized and the surgical field re-exposed. The monofilament suture was withdrawn and tied off on the ECA. The temporary suture on the CCA was removed to allow blood recirculation. The skin was closed with an autoclip or a 4–0 silk suture. The mice were placed under the heating lamp on the heating plate for 0.5 h. After checking that the mice regained mobility, the mice were returned to the cage. At 6, 24 and/or 72 h after the induction of tMCAO for 0.5 h, biological and histological analyses were performed as follows.

The hemolymph pH in vivo was measured using a needle-type fiber-optic pH microsensor (tip size ca. 140 μm) connected to a PreSens pH 1 micro-detection device according to the manufacturer’s instructions (PreSens Precision Sensing GmbH, Regensburg, Germany). The pH probe was calibrated at 25 °C with standard pH 4.01, 6.86, 7.41, and 9.18 buffer solutions. The pH microsensor was mounted on the manipulator of a stereotaxic apparatus (Narishige, Tokyo, Japan). After the induction of tMCAO (0.5 h) and permanent MCAO (0.5–24 h), the mice were anesthetized with 2% isoflurane, and the head was fixed in the stereotaxic apparatus. Following skin incision, two holes corresponding to the ipsilateral and contralateral regions of interest were made in the skull using an electric drill. Anesthesia was maintained via inhalation of 1.5% isoflurane. Localized pH changes were measured with the pH microsensor in the ipsilateral and contralateral regions (ROIs; 1.5–2 mm lateral, 3–3.5 mm ventral and 1 mm anterior to the bregma based on the mouse brain atlas).

Laser Doppler flowmetry (ALF21 with BF04436, ADVANCE, Tokyo, Japan) was occasionally used to monitor cerebral blood flow during surgery for MCAO as described previously^49^. A small incision was made in the skin overlying the temporalis muscle, and the probe was fixed with instant glue on the superior portion of the temporal bone (6 mm lateral and 2 mm posterior to the bregma) as described previously^50^.

### Treatment of minocycline in vivo

Minocycline was used to assess microglial activation and function in the deterioration of ischemic injury at 24 h after the induction of tMCAO for 0.5 h and reperfusion. Minocycline hydrochloride dissolved in 5 mg/mL (10 mmol/L) saline or vehicle was administered intraperitoneally at 50 mg/kg once daily beginning 3 days before and immediately after the surgery.

### Neurological scores

Neurological deficits were scored as previously described^35^. Behavioral assessments were made 24 h after the induction of tMCAO for 0.5 h and reperfusion. Three independent blinded investigators graded the neurological scores. The neurological deficits were scored as follows: 0, normal; 1, mild turning behavior with or without inconsistent curling when picked up by tail, < 50% attempts to curl to the contralateral side; 2, mild consistent curling, > 50% attempts to curl to contralateral side; 3, strong and immediate consistent curling, mouse holds curled position for more than 1–2 s, the nose of the mouse almost reaches the tail; 4, severe curling progressing into barreling, loss of walking or righting reflex; 5, comatose or moribund.

### Tissue preparation for histology

The mice were perfused through the heart with saline and then 4% paraformaldehyde in phosphate-buffered saline (PBS) under deep anesthesia induced by sodium pentobarbital (60 mg/kg i.p.). The brains were removed and fixed at 4 °C in 4% paraformaldehyde in PBS for 24 h. For in situ hybridization, the brains were then immersed for more than 24 h in PBS containing 30% sucrose at 4 °C and then rapidly frozen with O.C.T. Compound (Sakura Finetek Japan, Tokyo, Japan). For embedding tissue into paraffin blocks, the tissues were dehydrated with an ascending ethanol series and then immersed in xylene and embedded in paraffin. Serial sections equivalent to the coronal brain slice 1 mm anterior to the bregma at 5 μm intervals were mounted on slides. The histological images were analyzed independently by blinded investigators and technical staff using differentiated symbols and numbers for mice and tissue samples. The results were later compared with the corresponding histology.

### In situ hybridization and immunohistochemistry

For the analysis of TDAG8 mRNA expression in mouse brains, serial coronal sections from the frozen blocks were prepared as describe above. The cDNA fragments of TDAG8 were obtained by PCR as follows: TDAG8_475 (475 bp in GenBank NM_008152) from the total RNA of mouse cultured microglia with 5′-ATCCCTCCAGAAACAGGGAAACATG-3′ and 5′-TCTTCAATGCACATGCTGTTCATCG-3′ and subcloned in sense or antisense orientation into the pCR2.1 vector (Life Technologies, Carlsbad, CA, USA). The digoxygenin (DIG)-labeled riboprobes were produced using these plasmids as templates for in vitro transcription (Roche Diagnostics GmbH, Mannheim, Germany). Hybridization was performed essentially as described previously^51^. Briefly, the frozen sections were incubated for 16 h at 50 °C in 20 ng/mL of each DIG-labeled RNA probe in a hybridization buffer (Nippon Gene, Tokyo, Japan). The sections were treated with RNase A and then washed twice with 0.2 × standard saline citrate (SSC) for 15 min. The sections were immersed in 1.5% blocking reagent (Roche Diagnostics GmbH, Mannheim, Germany) for 1 h at 37 °C. The sections were then incubated with an alkaline phosphatase-conjugated anti-DIG antibody (Roche Diagnostics GmbH, Mannheim, Germany) diluted to 1:2000 in the same buffer for 16 h at 4 °C. After being washed with the same buffer, the sections were treated with a chromogen solution (0.34 mg/mL nitro blue tetrazolium (NBT), 0.18 mg/mL 5-bromo-4-chloro-3-indolylphosphate p-toluidine salt (BCIP)) until a visible signal was detected. There were no apparent signals in the control sections according to the sense probes. The sections were also used for immunostaining to locate Iba1-positive cells with anti-Iba1 antibody (1:400 dilution). The positive signals were detected by an anti-rabbit Alexa Fluor 488 conjugated secondary antibody (1:1000 dilution). After processing, the sections were mounted and examined by fluorescence light microscopy.

### Nissl staining

To measure the infarct area of the coronal Sect. 1 mm anterior to the bregma, serial coronal sections were prepared from the paraffin blocks as described above. The sections were deparaffinized with xylene, rehydrated through descending concentrations of ethanol, and washed in water. The sections were then stained with 0.1% cresyl violet and washed in 95% and 100% ethanol until the background was nearly clear. After processing, the sections were cleared in xylene and mounted with Entellan. Images were acquired using a 4 × objective lens under a bright-field microscope (KEYENCE BZ-9000 BioRevo). The areas of infarction were delineated and quantified using ImageJ software and the infarct (%) was calculated based on the lesion areas in the ipsilateral hemisphere.

### TTC staining

Brains were removed under deep anesthesia as described above (sodium pentobarbital, 60 mg/kg i.p.). The brains were cut in 2 mm coronal sections, immersed in a 2% solution of TTC dissolved in saline and stained for 20 min at 25 °C in the dark. The stained brain tissue was fixed in 4% formalin in PBS. The image was captured with the scanner and unstained lesion areas were measured using ImageJ software, and the infarct (%) was calculated based on the lesion areas in both the anterior and posterior sides for each slice of the ipsilateral hemisphere.

### Magnetic resonance imaging (MRI)

MRI was performed using an ICON MRI Scanner (Bruker Biospin K.K. Kanagawa, Japan) at the Bioresource Center of Gunma University Graduate School of Medicine. After the induction of tMCAO, the mice were anesthetized with 2% isoflurane and put securely in position in the animal holder. The mice were monitored using a respiration system, and anesthesia was maintained by the inhalation of 1.5% isoflurane. The body temperature of the mice was maintained with a heating system. The imaging protocol for head anatomy included a T2 RARE highres and a T1 RARE (ParaVision 5.1). The infarction volume was extracted from the T2 RARE highres image (2 × 7 slices, 1 mm thickness and 1 mm gap). The regions of interest (ROIs) were configured over regions of the lesion as defined by T2-weighted MRI for each slice, and the infarct volume (mm^3^) was calculated by summing the lesion areas of all slices and integrated by the slice thickness using OsiriX Lite.

### RT-qPCR

Total RNA was prepared from the cerebral hemisphere according to the manufacturer’s instructions for RNAisoPlus (Takara, Japan). RT-qPCR was performed using TaqMan hydrolysis probes (Applied Biosystems) as described previously^52^. The total RNA (5 μg) was treated with DNase I to remove possible traces of genomic DNA and subjected to RT-qPCR. The thermal cycling conditions were as follows: 2 min at 50 °C, 10 min at 95 °C, 40 cycles of 15 s at 95 °C, and 1 min at 60 °C. The expression level of the target mRNA was normalized to the relative ratio of the expression of GAPDH mRNA. The RT-qPCR assay was performed with three different RNA concentrations in each sample.

### Preparation of microglia and astrocytes and evaluation of cellular activities

Mouse astrocytes (evaluated with anti-GFAP antibody) were prepared as described^16^. Briefly, the cerebral cortex from 1- to 2-day-old mouse pups was minced and digested with 0.25% trypsin for 20 min at 32 °C. Dissociated cells were collected, resuspended, and filtered (71 μm) in Dulbecco’s modified Eagle’s medium (DMEM) containing 10% fetal bovine serum (FBS), and then plated at a density of 1.0 × 10^6^ cells/mL on a poly-d-lysine-coated flask (75 cm^2^). The cultures were maintained for 20 days until confluent. The growth medium was collected and stored as a glial conditioned medium containing 10% FBS (G-DMEM). Microglia were prepared using a mild trypsinization method as described previously^53^. Briefly, the cell suspension obtained by mincing and digestion with 0.25% trypsin of the cerebral cortex as described above was plated on regular culture dishes. The adherent cells are incubated in 0.05% trypsin (Trypsin 25200 from Invitrogen-GIBCO diluted in DMEM) for 30–60 min at 37 °C to remove astrocytes. The attached microglia were recovered using 0.25% trypsin for 10 min at 37 °C, resuspended in G-DMEM, and filtered (40 μm). The cell suspension was plated at a density of 3–5 × 10^5^ cells/mL in 6- or 12-well dishes for following experiments and cultured for 1 day. The resulting population consisted of > 95% microglia evaluated by anti-Iba1, anti-CD11b and anti-F4/80 antibodies. To assess the effect of minocycline on the LPS-induced TNF-α, IL-1β, and VCAM-1 production in microglia or astrocytes, the cells were cultured on 6-well plates, pre-treated with 30 μmol/L minocycline at 37 °C in the culture medium for 24 h, and serum-starved in a fresh DMEM containing 0.1% BSA and 30 μmol/L minocycline for 8 h. The cells were then stimulated for 16 h with 10 ng/mL LPS in the DMEM. Microglia were also plated on 12-well plates for the analysis of TNF-α and IL-1β production. The culture medium was changed to fresh DMEM containing 0.1% BSA for 8 h. The dishes were then stimulated for 16 h with HEPES-buffered α-minimum essential medium (MEM) containing 20 mmol/L HEPES, 0.1% BSA and 1 μg/mL LPS under an appropriate pH.

### Estimation of TNF-α, IL-1β, pro-IL-1β, VCAM-1, and actin by Western blot analysis

The incubation medium from 6-well plates after the LPS stimulation was collected by centrifugation at 14,000 g for 1 min. The medium was then concentrated approximately 10 times by Ultracel-3K (Merck Millipore Ltd., Darmstadt, Germany) and stored at − 80 °C until Western blot analysis. For detection of the cytoplasmic precursor of IL-1β (proIL-1β), VCAM-1, and actin, the cells were washed twice with ice-cold PBS and harvested from the dishes with a rubber policeman by adding a lysis buffer composed of PBS, 1% IGEPAL, 0.5% sodium deoxycholate, 0.1% SDS, 1 mmol/L EDTA, and 1% proteinase inhibitor cocktail (Sigma-Aldrich). The lysate was incubated for 30 min on ice and was centrifuged at 14,000×*g* for 20 min. The protein concentration of extracts was determined with a BCA Protein Assay. The concentrated medium and recovered lysate were subjected to 12.5% SDS–polyacrylamide gel electrophoresis and analyzed by Western blotting with primary antibodies. The membranes were then incubated with a second antibody conjugated with alkaline phosphatase and the blots were visualized using the NBT/BCIP system as described previously^16^. The expression level of the target protein was normalized to the relative ratio of actin.

### Measurement of TNF-α and IL-1β in the medium using ELISA

The HEPES-buffered medium from 12-well plates after LPS stimulation was collected by centrifugation at 14,000×*g* for 1 min. The pH in the sample was adjusted to around 7.4 by the addition of 0.5 mol/L HCl or NaOH and stored at − 80 °C until evaluation of the cytokine content. A commercially available ELISA kit was used to determination of the TNF-α and IL-1β concentration according to its instruction manual. The expression level of the target protein was normalized to the relative ratio of the cell-protein lysate as described above.

### Statistical analysis

GraphPad Prism 6 (La Jolla, CA, USA) was used for the statistical calculation. For experiments in vivo, more than two mice per group in each experiment were employed for the same experiments at least three times and the results were combined for the presentation unless otherwise stated. The results are presented as the mean ± SEM. Student’s *t*-test for two-group comparisons and a one-way ANOVA followed by the Tukey test were used to determine differences between the control and experimental groups, and a two-way ANOVA followed by the Tukey test was used to determine the differences between multiple-group comparisons: values were considered significant at *p* < 0.05 or *p* < 0.01. For neurological scores, the unpaired Mann–Whitney test was used to assess statistical significance. For experiments in vitro, the results of multiple observations are presented as the mean ± SEM or as representative results from more than three different experiments. Statistical significance was assessed using the Multiple *t*-test (Holm-Sidak method); values were considered significant at *p* < 0.05.

## Supplementary information

Supplementary file1
