# Supplementary material for: The protective role of proton-sensing TDAG8 in the brain injury in a mouse ischemia reperfusion model
Source: Sci Rep. 2020 Oct 14;10:17193. doi: 10.1038/s41598-020-74372-7 (PMC7566628; doi:10.1038/s41598-020-74372-7)
Supplement: Supplementary file 1 — Supplementary file1 [file 41598_2020_74372_MOESM1_ESM.pdf]

Supplementary information

The protective role of proton-sensing TDAG8 in the brain injury in a mouse ischemia reperfusion model

Koichi Sato[1,*], Ayaka Tobo[1], Chihiro Mogi[1], Masayuki Tobo[1], Nobuhiro Yamane[2], Masahiko Tosaka[2], Hideaki Tomura[3], Dong-Soon Im[4], Fumikazu Okajima[5]

a

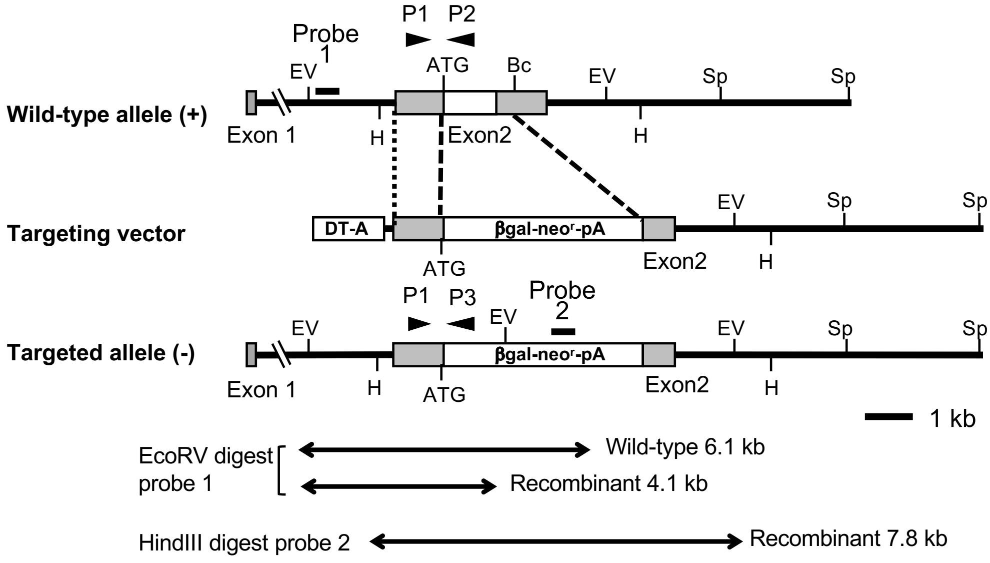

b

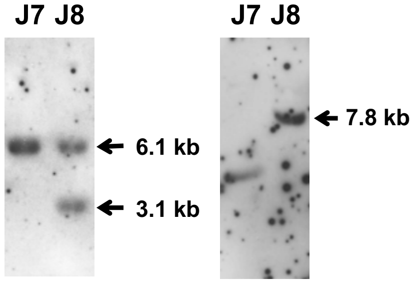

c

Primers used for genotyping

P1	TGTGTAGATAGCCCATTGACATTAGTTCCA
P2	CAGGTCTGCAATGCTCAAGTTCATC
P3	CATTCGCCATTCAGGCTGCGCAACTG

Supplementary Figure S1. Generation of *gpr4* deficient mice. (**a**) Restriction maps of mouse *gpr4* wild-type allele, targeting vector, and targeted allele. The cassette containing β-galactosidase/neomycin phosphotransferase fusion gene was subcloned into the targeting vector replacing the *gpr4* coding sequence in exon 2. EV, Eco RV; H, Hind III; Sp, Spe I; Bc, Bcl I. (**b**) Southern blot analysis of targeted ES cell clones. Genomic DNA from homologous recombinant clone (J8) or non-homologous clone (J7) were digested with Eco RV for hybridization with probe 1 (left) or with Hind III for hybridization with probe 2 (right). (**c**) PCR primers that were used to characterize the targeted *gpr4* gene are shown.

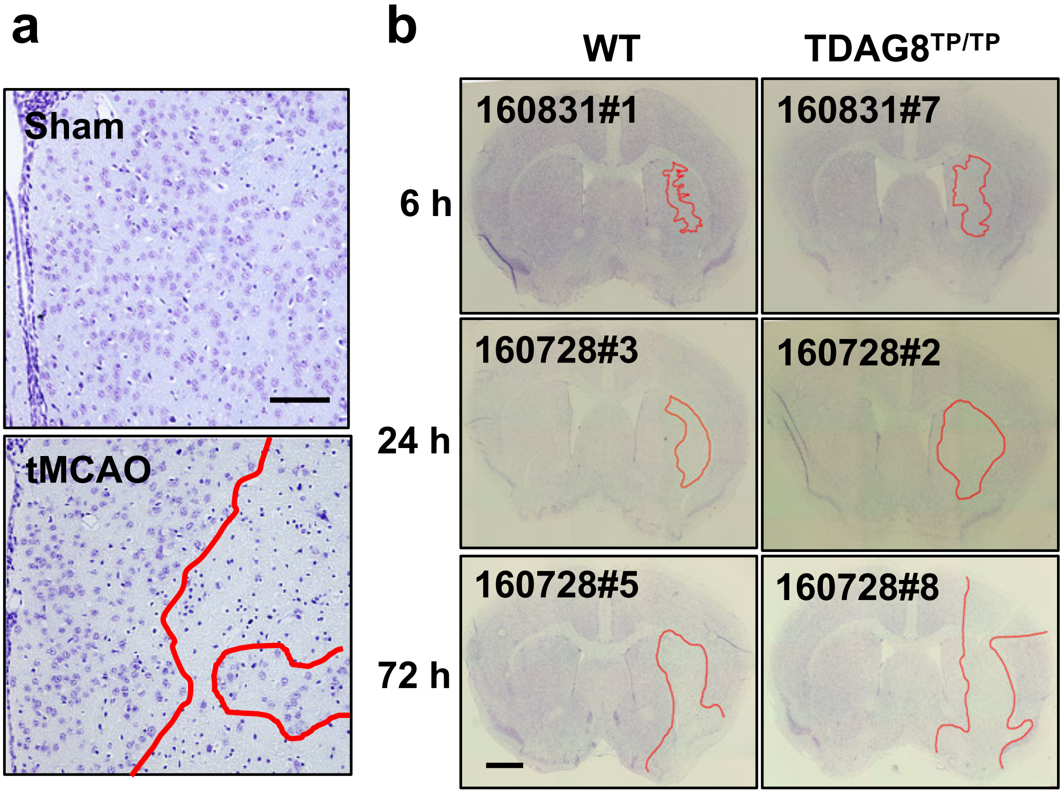

Supplementary Figure S2. The MCA occlusion leads to an injury in the striatum and frequently in parts of the neocortex at 1 mm anterior to bregma. (**a**) Nissl staining in the striatum close to the lateral ventricle after tMCAO for 0.5 h and reperfusion for 24 h. The tMCAO induced appreciable cell damage compared to the sham surgery. Red lines indicate the infarct regions with cell damage. Scale bar is 100 µm. Section images are representatives of more than three different batches. (**b**) Time course study for induction of infarction after tMCAO for 0.5 h in wild-type mice (WT) and TDAG8 mice (TDAG8$^{Tp/Tp}$). In the striatum irregular spotted impairments were induced 6 h after reperfusion. The parts of striatum contributed to cerebral infarct size after reperfusion for 24 h. Following the reperfusion for 72 h, impaired cell damage zones include neocortex. Scale bar is 1.2 mm. Section images are representatives of two different batches.

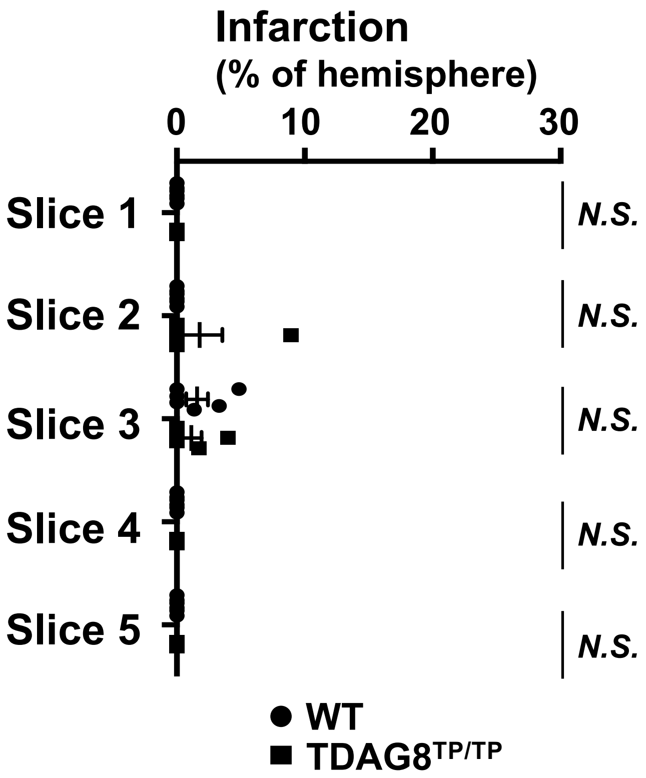

Supplementary Figure S3. Examination of stroke injury one month post-tMCAO for 0.5 h and reperfusion in mouse brain. Unstained infarction was obtained by analyses of coronal sections stained with TTC in WT mice (closed circle, n = 6) and TDAG8$^{TP/TP}$ mice (closed square, n = 5). The infarction (%) was calculated by the lesion areas of the ipsilateral hemisphere. Error bars represent mean ± SEM. Comparisons between WT and TDAG8 $^{TP/TP}$ mice were assessed using the unpaired Student's *t*-test. (N.S., not significant).

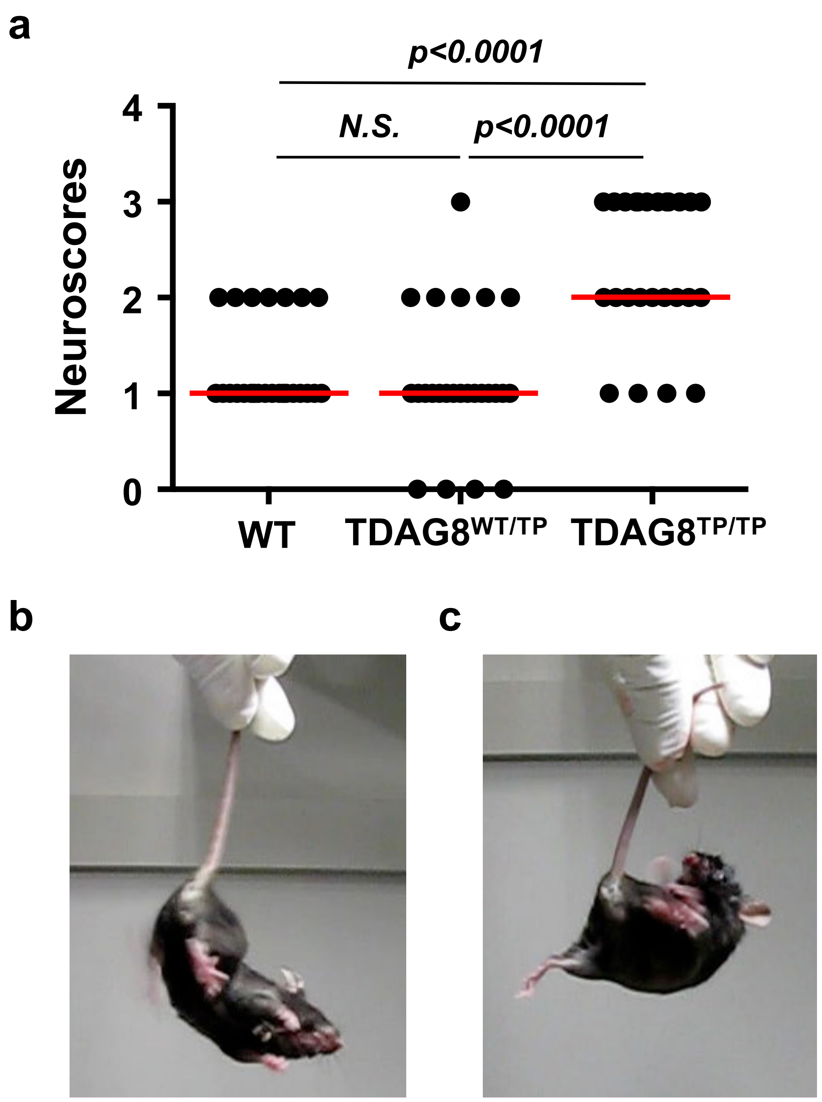

Supplementary Figure S4. The exacerbation of neurological scores by the TDAG8 deficiency. (**a**) After WT (n = 25), TDAG8$^{WT/Tp}$ (heterozygous, n = 25) and TDAG8$^{Tp/Tp}$ mice (homozygous, n = 25) were subjected to tMCAO for 0.5 h and reperfusion for 24 h, neurological deficits are assessed by scoring the extent with 6 levels (0 to 5) based on the behavioral function as described in Methods. Data are shown as neurological scores (closed circle) and median (red line). Comparisons among groups were assessed using the unpaired Mann-Whitney test (N.S., not significant). Representative pictures for WT mice with level 1 (**b**) and level 3 (**c**) are shown.

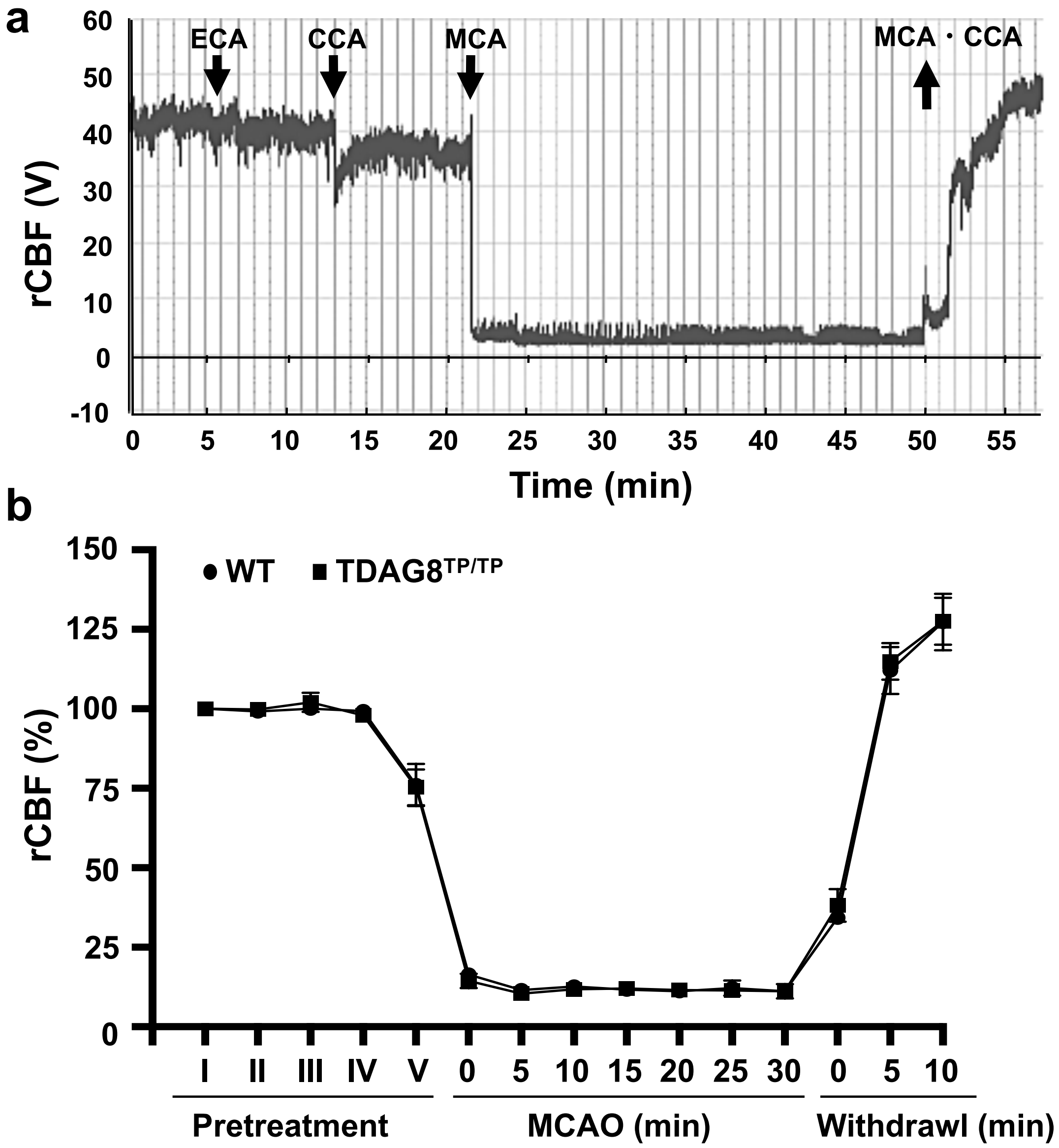

Supplementary Figure S5. Lack of influence by TDAG8 deficiency in regional cerebral blood flow of the MCA area due to ischemia. (**a**) Typical regional cerebral blood flow (rCBF) trace for the occlusion of the MCA. The arrows indicate ligation of the ECA, temporary occlusion of the CCA, the occlusion of MCA, and reperfusion of MCA (withdrawal of the filament). (**b**) The change in rCBF in WT (closed circle, n = 6) and TDAG8$^{Tp/Tp}$ mice (closed square, n = 6) was shown as the means ± SEM. The results were expressed as percentages of each basal value. Pre-treatment indicates: I, basal value; II and III, before and after ligation of the ECA; IV and V, before and after occlusion of the CCA. Comparisons between WT and TDAG8 $^{TP/TP}$ mice were assessed using the unpaired Student's *t*-test, and the effect of the TDAG8 deficiency was not significant.

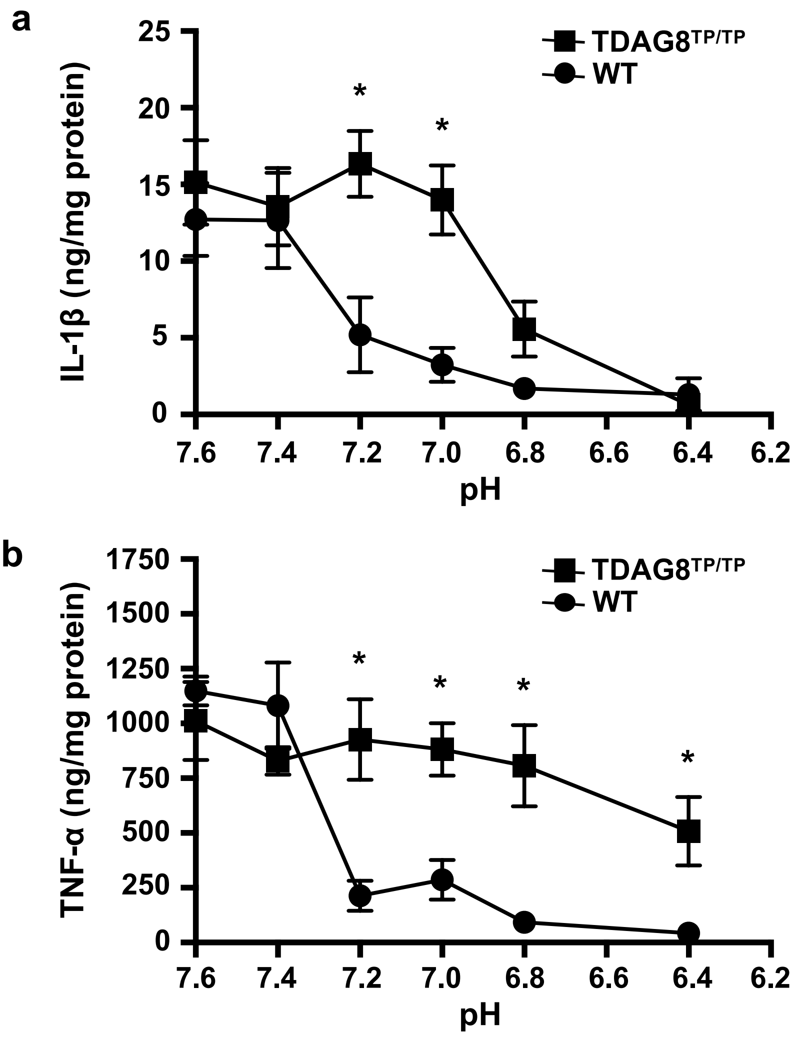

Supplementary Figure S6. Effects of the TDAG8 deficiency on the inhibitory effect of extracellular acidic pH-induced IL-1β and TNF-α production in mouse isolated microglia. (**a, b**) The cells from WT mice (open symbol) and $TDAG8^{Tp/Tp}$ mice (closed symbol) were serum-starved in DMEM medium containing 0.1% BSA for 8 h, and incubated at 37°C in HEPES-buffered α-MEM with indicated pH containing 1 μg/mL LPS for 16 h. The IL-1β (**a**) and TNF-α (**b**) contents were measured in the incubation medium by an ELISA method, and are expressed as pg/mg protein of the adherent cells. Data are shown as the mean ± SEM of three separate experiments in duplicate. Comparisons among groups were assessed using the Multiple *t*-test. The asterisk indicates that the effect of the TDAG8 deficiency was significant in cytokine contents ($*p < 0.05$).

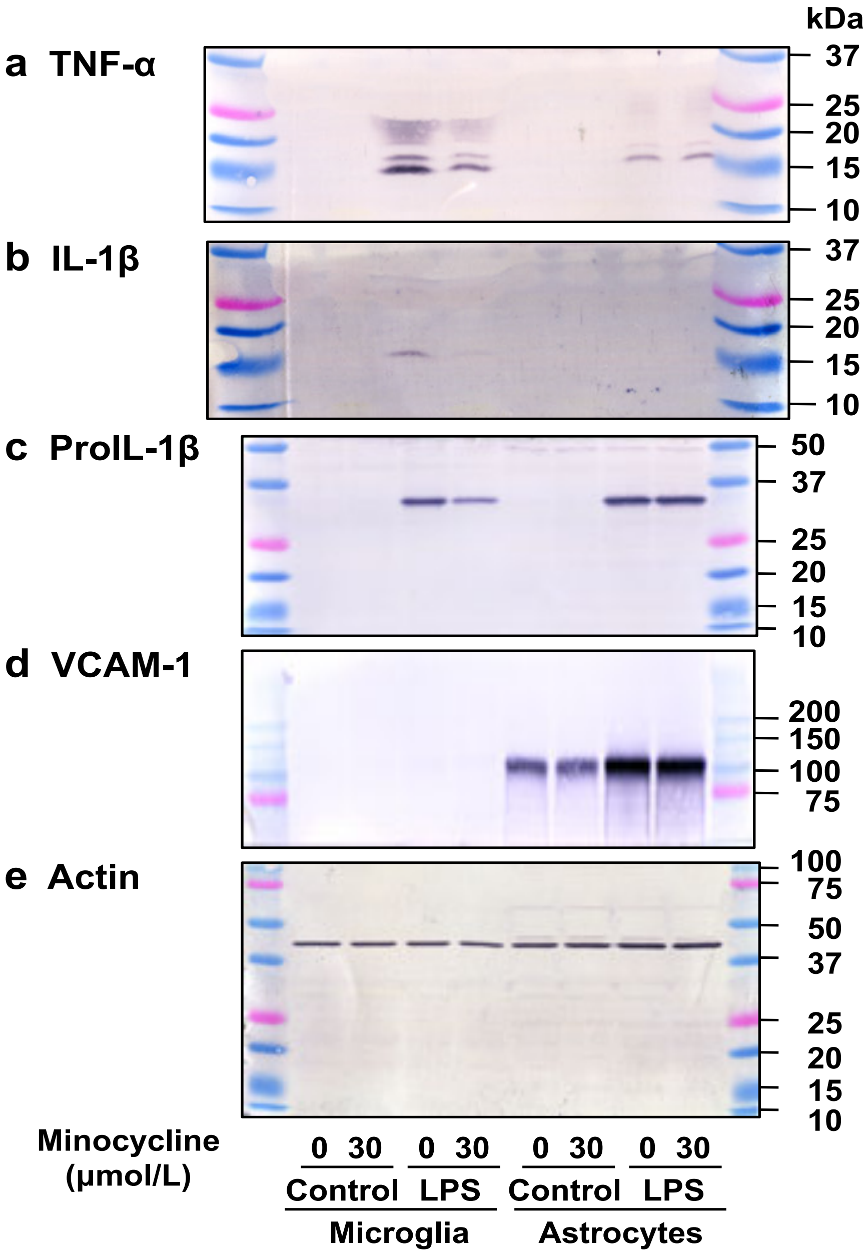

Supplementary Figure S7. Effects of minocycline on the expression of cytokines and an adhesion molecule in isolated microglia and astrocytes. (**a-e**) Microglia and astrocytes were pre-treated with 30 μmol/L minocycline at 37 °C in the growth culture medium for 24 h. The cells were incubated with 30 μmol/L minocycline in the serum-starved DMEM containing 0.1% BSA for 8 h and further incubated with 10 ng/mL LPS in the same culture medium for 16 h. The IL-1β (**a**) and TNF-α (**b**) were measured in the culture medium and proIL-1β (**c**), VCAM-1 (**d**), and actin (**e**) were in cell lysates by the Western blotting. All blots are photocopies obtained from different gels with an aliquot of each sample. Data are representatives of three separate experiments.

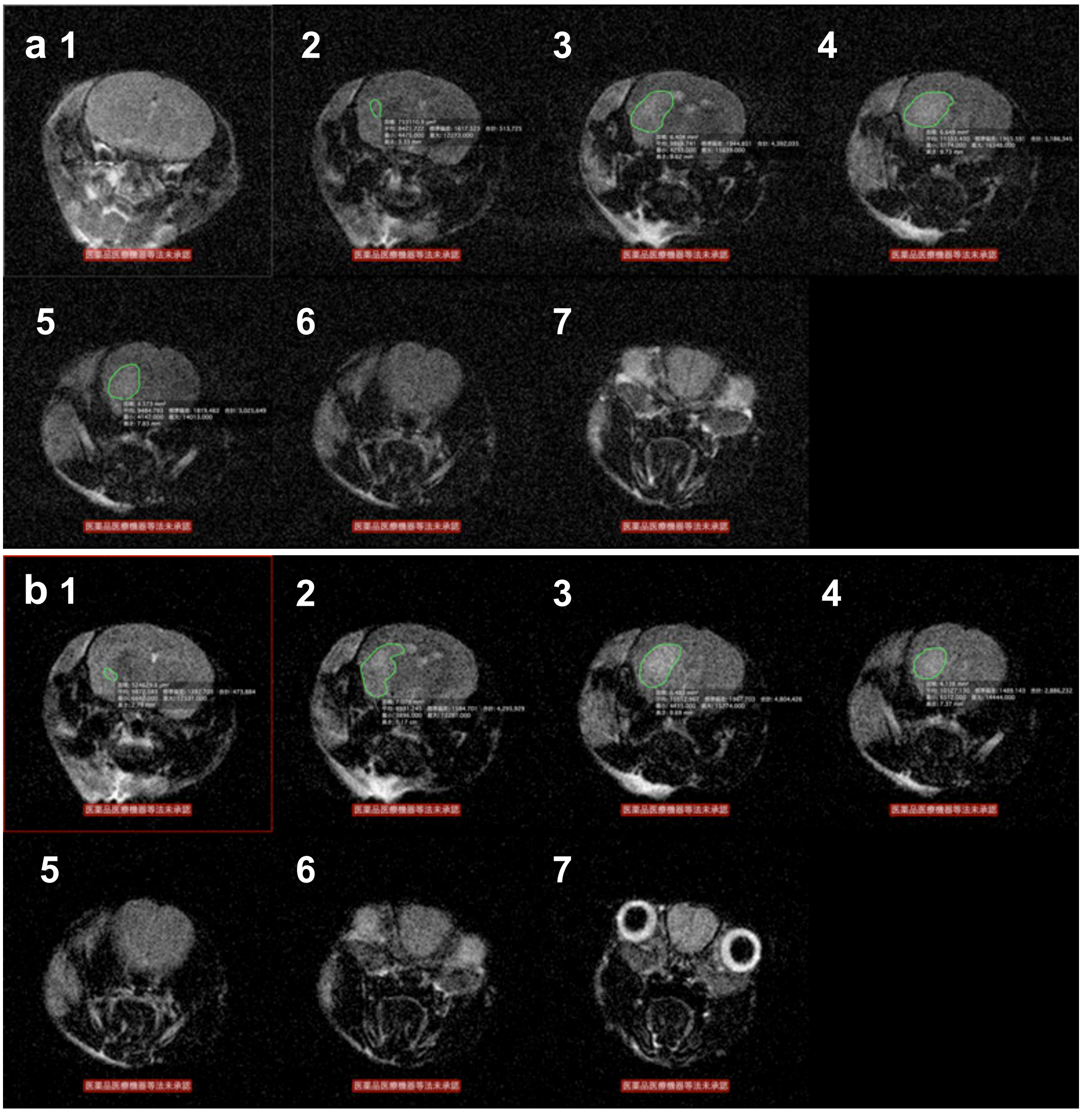

Supplementary Figure S8. Representative images of Magnetic Resonance Imaging (MRI) for a mouse brain 24 h after tMCAO for 0.5 h and reperfusion. (**a, b**) The lesion can be assessed using T2-weighted images with the ipsilateral region in comparison with the contralateral region. The T2 images were obtained twice by 7 slices with 1 mm thickness and 1 mm gap. The position of the lesion is shown by the green line in (**a**) 2-5 and (**b**)1-4. The regions of interest (ROIs) was configured over the lesion in each slice, and infarct volume (mm3) was calculated.
